# Unsuspected Rickettsioses among Patients with Acute Febrile Illness, Sri Lanka, 2007

**DOI:** 10.3201/eid1805.111563

**Published:** 2012-05

**Authors:** Megan E. Reller, Champica Bodinayake, Ajith Nagahawatte, Vasantha Devasiri, Wasantha Kodikara-Arachichi, John J. Strouse, Judith E. Flom, Truls Østbye, Christopher W. Woods, J. Stephen Dumler

**Affiliations:** Johns Hopkins University School of Medicine, Baltimore, Maryland, USA (M. E. Reller, J.J. Strouse, J.S. Dumler);; Johns Hopkins School of Public Health, Baltimore (J.E. Flom);; Medical Faculty of University of Ruhuna, Galle, Sri Lanka (C. Bodinayake, A. Nagahawatte, V. Devasiri, W. Kodikara-Arachichi);; Duke University School of Medicine, Durham, North Carolina, USA (T. Østbye, C.W. Woods)

**Keywords:** Rickettsial infection, rickettsia, serologic tests, Sri Lanka, fever, scrub typhus, bacteria

## Abstract

We studied rickettsioses in southern Sri Lanka. Of 883 febrile patients with paired serum samples, 156 (17.7%) had acute rickettsioses; rickettsioses were unsuspected at presentation. Additionally, 342 (38.7%) had exposure to spotted fever and/or typhus group rickettsioses and 121 (13.7%) scrub typhus. Increased awareness of rickettsioses and better tests are needed.

Globally, rickettsioses are increasingly recognized as causes of undifferentiated fever. Paired serum samples are infrequently obtained, but testing acute-phase serum alone is insensitive (IgG is initially absent) and nonspecific (IgG can persist for years, and IgM results represent cross-reactions).

Sentinel studies in Malaysia (*1*), Thailand (*2*), India (*3*), Laos (*4*), and Nepal (*5*) suggest that scrub and murine typhus are frequent and that misdiagnosis as enteric fever results in ineffective therapy (*5*). Unrecognized rickettsial species are likely present in Sri Lanka, an island connected to the southern tip of India by an underwater 30-km land bridge. Kularatne reported acute rickettsioses diagnosed by using only acute-phase serum IgM in 56 of 118 patients who had fever in hilly central Sri Lanka (*6*); another study in the Western Province confirmed few (5/31cases) of suspected rickettsioses (*7*). Both studies were limited by selective enrollment. To characterize rickettsioses among undifferentiated febrile illnesses in southern Sri Lanka, we prospectively studied patients who came to a large hospital.

## The Study

Consecutive patients >2 years of age with fever (>38°C tympanic) who came to Teaching Hospital Karapitiya were enrolled (*8*). Standardized epidemiologic and clinical data and blood were obtained during acute illness and 2–4 weeks later. During the study (March–October 2007), the atmospheric temperature ranged from 27.5°C–32°C (high) to 24°C–26°C (low), and rainfall was variable (mean 301 mm/mo, range 36–657 mm/mo).

Because rickettsial species broadly cross-react within groups (*9**,**10*), paired serum samples were tested by using an IgG indirect immunofluorescence assay (IFA) and *Rickettsia rickettsii* and *R. typhi* antigens (Focus Diagnostics, Cypress, CA, USA) to identify infections with spotted fever group (SFGR) and typhus group (TGR) rickettsial infections. Serum samples reactive at a titer of 80 were considered potentially positive and were titered.

To identify scrub typhus (ST) infections, we tested paired serum samples using IgG ELISA as described (*11*), except for use of recombinant antigens (0.2 µg each of r56 Chimeric1, Gilliam, and Kato strains) to detect antibodies to *Orientia tsutsugamushi*. Comparative blind testing of 200 serum samples with an established (pooled-antigen) quantitative assay enabled validation (*12*).

Acute rickettsioses (SFGR, TGR, and ST) required a >4-fold rise in specific IgG titer or its equivalent; patients with equal SFGR and TGR convalescent-phase titers were SFGR/TGR group-indeterminate. IgG (titer >160) in acute-phase serum defined rickettsial exposure (seroprevalence). Stata IC version 11.0 (StataCorp LP, College Station, TX, USA) was used for analyses.

We analyzed paired serum samples for rickettsioses for 883 (81.9%) of 1,079 patients. Median acute–convalescent phase follow-up was 21 days (intraquartile range 15−33 days). Patients with and without paired serum samples were comparable (*8*). Acute rickettsioses were documented in 156 (17.7%) patients (Table 1). The increase in convalescent-phase geometric mean titer was 14-fold (845) for SFGR, 17-fold (920) for TGR, and 11-fold (951) for SFGR/TGR rickettsiae.

**Table 1:** Clinical and laboratory characteristics of febrile patients with and without acute rickettsial infections, southern Sri Lanka, 2007*

Characteristic	Rickettsial infection						
	None, n = 727	SFGR, n = 86	TGR, n = 29	*Orientia tsutsugamushi,* n = 9	SFGR/TGR, n = 24	SFGR or TGR *+ O. tsutsugamusi,* n = 8	p value†
Median age, y (IQR)	28 (18–47)	31 (22–46)	44 (21–59)	37 (19–58)	39 (22–48)	37 (22–56)	0.06
Male sex	86	8	2	0.3	2	1	0.10
Admitted to hospital	71	78	69	89	71	88	0.51
Symptoms							
Headache	78	79	79	67	79	63	0.86
Sore throat	29	28	21	22	29	13	0.82
Cough	60	49	54	56	30	63	0.048
Dyspnea	18	8	21	22	29	25	0.14
Joint pain	44	41	52	44	52	57	0.83
Muscle pain	48	52	57	56	42	43	0.83
Fatigue	66	69	66	56	71	63	0.96
Abdominal pain	19	19	21	33	30	0	0.43
Emesis	37	36	34	44	54	43	0.65
Diarrhea	12	9	7	22	13	0	0.66
Dysuria	13	23	7	22	25	13	0.07
Oliguria	8	17	3	11	21	13	0.02
Signs							
Conjunctivitis	13	21	24	22	13	50	0.008
Throat exudate	15	12	21	0	8	0	0.41
Lymphadenopathy	23	20	14	22	21	38	0.74
Jaundice	2	0	4	0	0	0	0.70
Pulmonary rales	15	5	14	22	8	0	0.09
Tender abdomen	10	8	10	0	17	0	0.63
Hepatomegaly	5	6	3	22	0	13	0.19
Rash‡	9	15	0	50	0	0	0.18
Laboratory measures, mean ± SD							
Leukocytes, cells/μL	8,893 ± 4,634	8,253 ± 3,774	10,341 ± 7,931	9,778 ± 4,800	9,129 ± 3,925	9,943 ± 4,527	0.96
ANC, cells/μL	6,325 ± 4,054	5,825 ± 3,263	7,114 ± 4,905	7,280 ± 3,890	6,494 ± 3,887	6,514 ± 3,762	0.57
ALC, cells/μL	2,317 ± 1,234	2,284 ± 1,378	2,822 ± 3,170	2,380 ± 1,291	2,460 ± 1,215	3,036 ± 1,526	0.33
Hemoglobin, g/dL	12.6 ± 1.7	12.6 ± 1.5	12.7 ± 1.6	13.0 ± 2.2	12.7 ± 1.6	12.4 ± 2.0	0.98
Platelets × 1,000/μL	249 ± 99	228 ± 84	237 ± 81	187 ± 74	219 ± 72	301 ± 119	0.05

*Values are % patients except as indicated. SFGR, spotted fever group rickettsiae; TGR, typhus group rickettsiae; IQR, intraquartile range; ANC, absolute neutrophil count; ALC, absolute lymphocyte count. †Kruskal-Wallis test to compare proportions and non-normally distributed continuous variables. Analysis of variance test to compare normally distributed continuous variables. ‡n = 309; 6/6 with rickettsioses and rash had eschars, compared with 11/22 of those without rickettsioses (p<0.001).

**Figure 1:**
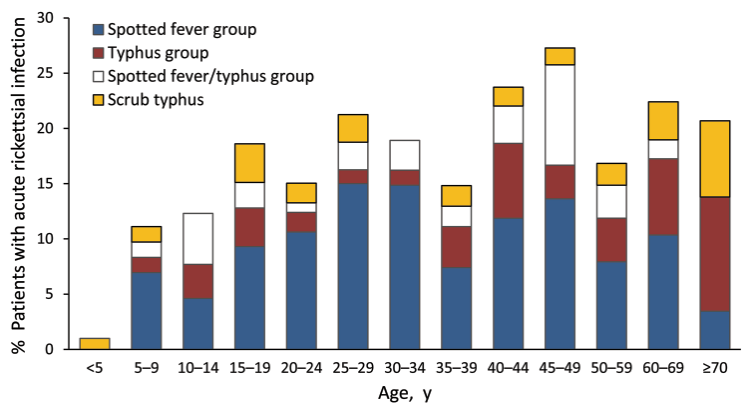
Proportion of febrile patients with acute rickettsial infections by age group, southern Sri Lanka, 2007.

**Figure 2:**
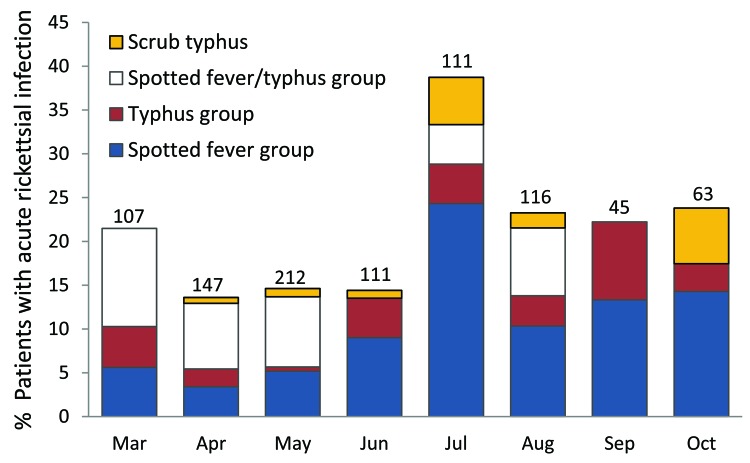
Proportion of febrile patients with acute rickettsial infections by month, southern Sri Lanka, March–October 2007.

Acute rickettsioses were found in 19.7% of patients >18 years of age and 10.5% of patients <18 years of age (p = 0.003); patients with rickettisoses were older than those without rickettsioses (median age 34.5 vs. 28.0 years; p = 0.005) (Figure 1). Among patients <18 years of age, acute rickettsioses were more common in male than in female patients (14.6% vs. 5.8%; p<0.05). Patients with acute ST alone were older than those with other rickettsioses and those without rickettsioses (median 36.5 vs. 34.4 vs. 28.0 years; p = 0.02) and more likely to report rice paddy exposure (44.4% vs. 15.1% vs. 9.3%; p = 0.001). More acute rickettsioses occurred during the July–October rains (70.6% of ST infections and 59.7% of other rickettsioses), whereas more nonrickettsial infections (67.0%) occurred during the drier period of March–June (p<0.0001) (Figure 2).

Acute rickettsioses were clinically similar to each other and to nonrickettsial febrile illnesses, except for frequency of cough, oliguria, and conjunctival suffusion (Table 1). Except for a higher temperature with SFGR than with TGR infection (mean 38.6 vs. 38.2°C; p = 0.006), no feature differentiated these rickettsial infections. Conjunctival suffusion was more common (p = 0.004) with ST (35.3%) than with SFGR/TGR (12.8%) or no rickettsiosis (12.8%).

Antecedent antimicrobial drug use was commonly reported in patients with (45/115 [39.1%]) and without (195/536 [36.4%]; p = 0.58) rickettsioses. Amoxicillin and cephalosporins were administered most frequently in both groups (16.8% and 20.3%; p = 0.40), but infrequent administration of doxycycline (0.9% and 1.1%; p = 0.82) was recorded for 813 patients with paired serum samples, including 139 with acute rickettsioses. Rickettsioses were rarely clinically identified when present (3/139, sensitivity 2.2%, 95% CI 0.447%– 6.2%). Rickettsioses were also infrequently confirmed when diagnosed clinically (3 SFGR rickettsioses among 9 with suspected scrub typhus; positive predictive value 33.3%, 95% CI 7.5– 70.1) and rarely treated appropriately (2/9 given doxycycline, 1 with SFGR rickettsiosis). Patients with rickettsioses were hospitalized longer than others (median 5 vs. 4 days; p = 0.01), although the proportion hospitalized was similar. No one with confirmed rickettsioses died, but 11 of 12 patients died before follow-up.

At enrollment, 292 (33.1%) patients had IgG-confirmed rickettsial exposure. However, only 59 (20.2%) had a 4-fold increase in titer, and 97 (62.2%) of 156 acute infections were identified as rickettsioses by seroconversion. Exposures to rodents and rice paddies were associated with TGR rickettsiae and *O. tsutsugamushi* IgG (Table 2). Farmers were more likely (p = 0.01) to have IgG against *O. tsutsugamushi* (5.7%) than SFGR/TGR rickettsiae IgG alone (4.3%) or no rickettsial IgG (1.5%). If IgG titer >160 at either sampling were used to denote rickettsial exposure (preexisting or acute), 342 (38.7%) patients had exposure to SFGR and/or TGR rickettsiae. If a lower titer (>80) were used, 398 (45.1%) had such exposure. Similarly, 121 (13.7%) patients had either acute- or convalescent-phase IgG against *O. tsutsugamushi*.

**Table 2:** Epidemiologic characteristics of patients with and without IgG evidence of rickettsial exposure at enrollment, southern Sri Lanka, 2007

Characteristic	Negative rickettsial titer, n = 591	SFGR, n = 76	TGR, n = 12	*Orientia tsutsugamushi*,† n = 106	SFGR/TGR, n = 98	p value‡
Median age, y (IQR)	26 (16–44)	30 (23–48)	25 (19–48)	46 (34–56)	34 (23–55)	0.0001
Male sex	42	33	17	38	36	0.21
Residence						0.03
Urban	6	13	9	13	12	
Rural	94	87	91	87	88	
Type of work						<0.001
Homemaker	26	26	18	32	24	
Laborer	21	26	45	35	28	
Farmer	2	1	9	6	6	
Merchant	2	3	9	6	4	
Student	28	11	9	6	19	
Other	21	34	9	16	20	
Exposure						
Dog	57	46	50	57	44	0.10
Cat	35	26	25	30	30	0.43
Rodent	27	22	83	35	19	<0.001
Cow	5	4	17	5	6	0.48
Paddy field	10	8	0	18	8	0.07
Water source						0.03
Tap	29	45	17	29	35	
Boiled	13	1	8	8	4	
Well	57	54	75	65	60	
Other	1	0	0	1	1	

*Values are % patients unless otherwise indicated. SFGR, spotted fever group rickettsiae; TGR, typhus group rickettsiae; IQR, interquartile range. †Includes 8 patients with apparent SFGR or TGR co-infections. ‡Kruskal-Wallis test to compare proportions across groups.

## Conclusions

We documented endemic rickettsioses as a major cause of acute febrile illness in southern Sri Lanka. Epidemiologic features could not differentiate acute infection from prior exposure. Rickettsioses were infrequently suspected and not treated empirically when suspected. Underrecognition of rickettsioses could reflect nonspecific clinical features, limited diagnostic tools, lack of awareness that rickettsioses occur or cause severe illness, and absence of evidence-based local algorithms for acute febrile illness.

Studies in neighboring regions have used flawed methods, including spectrum bias, small sample size, selective enrollment, and testing acute-phase serum only. In many instances, clinical features were not predictive (*5**,**13*); however, older age was reported with rickettsioses (*14*) and lymphadenopathy and rapid respiratory rate with ST compared with TGR rickettsiosis (*4*). Although part of the rickettsial triad, rash is often absent initially (*10*) and among unselected febrile patients (*5**,**14*). SFGR/TGR cross-reactions and apparent co-infections could also impair clinical differentiation of specific rickettsioses. Laboratory abnormalities associated with ST (*3*) could reflect disease severity, not etiology, and such testing is often unavailable. Divergent conclusions might reflect different study populations, diagnostic criteria, reference groups, features evaluated, or real geographic differences.

Our estimate of rickettsioses is conservative. Confirmation required follow-up (return or home visit); the US Centers for Disease Control and Prevention no longer accepts a single high titer to confirm *R. rickettsii* infection (*10*). We required a 4-fold rise in titer even for seroconversions because IFA results are subjective, even among experts. We used *R. rickettsii* as a surrogate antigen, which could be less sensitive for detecting local SFGR. We chose ELISA for ST because a commercial IFA was not available and IFA as a standard for ST has been questioned (*15*).

Optimal clinical management of acute rickettsioses requires development of locally tested, evidence-based algorithms for acute febrile illness. Better diagnostic tests are needed to identify new species, elucidate vector–host relationships, and enable appropriate therapy. Molecular approaches hold promise but will require prospective validation.
